# Trigeminal–Facial Nerve Anatomical Connections and Their Clinical Value: A Narrative Review

**DOI:** 10.3390/diagnostics16121855

**Published:** 2026-06-15

**Authors:** Alexandra Diana Vrapciu, Alexia-Ioana Stancu, Victor Ioan Tibacu, Kyan-Tudor Zamani-Gavnani, Mugurel Constantin Rusu

**Affiliations:** Division of Anatomy, Faculty of Dentistry, Carol Davila University of Medicine and Pharmacy, 050474 Bucharest, Romania; alexia-ioana.stancu2024@stud.umfcd.ro (A.-I.S.); victor-ioan.tibacu2024@stud.umfcd.ro (V.I.T.); kyan-tudor.zamani2024@stud.umfcd.ro (K.-T.Z.-G.); mugurel.rusu@umfcd.ro (M.C.R.)

**Keywords:** dental anaesthesia, facial nerve, facial reanimation, muscle spindles, narrative review, perineural tumour spread, proprioception, sensorimotor integration, Sihler staining, synkinesis, trigeminal nerve, trigeminal–facial anastomosis

## Abstract

**Background/Objectives**: The trigeminal (CN V) and facial (CN VII) nerves are conventionally taught as separate pathways, yet extensive peripheral anastomoses form sensorimotor plexuses throughout the face. These communications provide the anatomical substrate for proprioception in facial muscles that paradoxically lack muscle spindles and Golgi tendon organs. This review aims to synthesise the anatomical, histological, and clinical evidence on these interconnections and to evaluate their implications across surgery, radiology, neurology, and dentistry. **Methods**: PubMed/MEDLINE, Scopus, and Google Scholar were searched for cadaveric dissection studies, Sihler whole-mount staining investigations, immunohistochemical analyses, quantitative axonal mapping studies, and clinical case series addressing trigeminal–facial communications and their diagnostic significance. **Results**: Twenty peripheral anastomoses were systematically identified and mapped, with prevalence ranging from reported-constant in multiple cadaveric series (auriculotemporal–facial trunk; mental–marginal mandibular) to variable (29–86%, depending on trigeminal division and method; V2 by cadaveric dissection, V1 by Sihler staining). Immunohistochemical evidence supports sensorimotor fibre interchange, and recent axonal mapping has revealed that the extracranial facial nerve is a mixed nerve containing motor, sympathetic, and afferent components. Clinically, these anastomoses are implicated in spontaneous facial recovery, trigeminal motor branch transfers, perineural tumour spread, local anaesthesia effects, synkinesis, and Ramsay Hunt syndrome. **Conclusions**: Available anatomical and histological evidence is consistent with the view that the trigeminal and facial nerves form a functionally integrated unit, though the functional significance of specific communications remains method-dependent. Recognition of these communications is relevant for surgeons, radiologists, neurologists, and dental practitioners managing facial conditions.

## 1. Introduction

The human face produces a wide range of expressions through precise coordination of sensory feedback and motor output, depending primarily on the trigeminal nerve (CN V) for facial sensation and the facial nerve (CN VII) for motor innervation of the muscles of expression [1,2]. Although often described as separate pathways, these nerves are extensively interconnected: terminal branches of CN V and CN VII form peripheral sensorimotor plexuses throughout nearly all facial regions [2,3,4,5]. The anatomical basis, histological nature, and clinical significance of these communications are the focus of this review.

Baumel [6] formally proposed that proprioceptive impulses from facial muscles—which lack classical muscle spindles—are conveyed centrally via trigeminal afferents communicating with facial nerve branches at the periphery. Since then, cadaveric studies by Namking et al. [7], Kwak et al. [8], Hwang et al. [9,10], and Tansatit et al. [11] have systematically mapped these communications, while whole-mount Sihler staining [12,13] and immunohistochemistry [4,14] have provided both topographic atlases and histological proof of genuine fibre interchange.

These connections are clinically relevant for surgeons operating on the parotid gland or performing nerve transfers for facial paralysis and face transplantation; for radiologists evaluating perineural tumour spread; and for clinicians managing hemifacial spasm, Ramsay Hunt syndrome, or the unintended effects of botulinum toxin and local anaesthesia [2,3,15].

Previous narrative reviews—notably Diamond et al. [3], focusing on clinical implications, and Cobo et al. [2], addressing the anatomical basis for facial muscle proprioception—provided the prior framework. However, neither integrated the quantitative axonal data of Tereshenko et al. [16,17], the refined Sihler criteria of Iwai et al. [18], nor the immunohistochemical mapping of Mohanty et al. [14]. The present review synthesises evidence from all four methodological traditions across the clinical domains in which CN V–CN VII communications carry documented consequences: facial reanimation and transplantation, parotid and midface surgery, dental anaesthesia, perineural tumour spread, and brainstem circuits. These domains were selected because they represent fields in which anatomical knowledge of the communications directly informs surgical planning, diagnostic interpretation, or pharmacological management.

## 2. Methods

This narrative review was conducted in accordance with the Scale for the Assessment of Narrative Review Articles (SANRA) [19]. The six SANRA criteria were met as follows: (1) scientific justification of the review question is provided in the Introduction; (2) the literature search is described below; (3) the evidence base consists of peer-reviewed primary studies; (4) the argumentation is structured by anatomical region and clinical domain; (5) the use of secondary literature is restricted to systematic reviews with pooled data; and (6) sources of potential bias, including methodological heterogeneity and self-citations, are explicitly acknowledged. The primary literature search was performed in PubMed/MEDLINE, Scopus, and Google Scholar from inception through March 2025 using combinations of the following terms: “trigeminal nerve,” “facial nerve,” “anastomosis,” “communication,” “proprioception,” “facial muscles,” “Sihler staining,” “perineural spread,” “facial reanimation,” “nerve transfer,” “buccinator,” “mental nerve,” “infraorbital nerve,” “auriculotemporal nerve,” “chorda tympani,” and “facial allotransplantation.” Boolean operators (AND, OR) linked anatomical terms with clinical and histological keywords. Reference lists of all retrieved articles were hand-searched to identify additional primary sources. Supplementary searches were conducted using the Consensus academic search engine, employing specific anatomical terminology, journal quality filters (Scimago Journal Rank ≤ 2), and publication year thresholds.

Representative PubMed search strings included: ((“trigeminal nerve”[MeSH] OR “facial nerve”[MeSH]) AND (“anastomosis” OR “communication” OR “nerve connection”) AND (“anatomy”[MeSH] OR “cadaver”[MeSH])); (“trigeminal”[tiab] AND “facial”[tiab] AND (“Sihler”[tiab] OR “nerve staining”[tiab])); and ((“perineural spread” OR “perineural invasion”) AND (“trigeminal” OR “facial”)). Equivalent strings were adapted for Scopus (TITLE-ABS-KEY). Google Scholar was queried using simplified keyword combinations; the first 200 results per query were screened. Only English-language publications were included. Hand-searching of reference lists yielded approximately 47 additional primary sources. The Consensus academic search engine was used as a supplementary retrieval tool for recent publications (2020–2025); the SJR rank was not applied as an eligibility criterion. Self-citations [20,21] are included solely where they constitute the primary source for specific anatomical data reported herein.

Studies were included if they reported original anatomical data (cadaveric dissection, whole-mount nerve staining, immunohistochemistry, or axonal morphometry), clinical case series or trials on trigeminal–facial interactions, neurophysiological evidence of trigeminal–facial reflex circuits, or imaging findings of perineural tumour spread. Systematic reviews providing pooled quantitative data were included; editorials and opinion pieces without original data were excluded. Because the included studies are heterogeneous in design, no formal meta-analysis was attempted; the evidence is presented as a structured narrative synthesis organised by anatomical region, histological method, and clinical domain.

Study characteristics (PICOS): Population—human subjects or cadaveric material; Intervention/Exposure—any procedure addressing CN V–CN VII communications; Comparators—not applicable; Outcomes—prevalence, morphometry, histological characterisation, or clinical outcomes involving these communications; Study types—cadaveric dissection, Sihler staining, immunohistochemistry, axonal mapping, clinical case series, controlled trials, neurophysiological studies, imaging studies, and systematic reviews with pooled data. Approximately 1200 records were identified after deduplication; 350 full-text articles were retrieved; 89 met the final inclusion criteria. Study heterogeneity—spanning cadaveric, histological, neurophysiological, and clinical designs—precluded formal meta-analysis; evidence is presented as structured narrative synthesis.

## 3. Overview of the Trigeminal and Facial Nerves

### 3.1. The Trigeminal Nerve (CN V)

The trigeminal nerve, the largest cranial nerve, arises from the pons and divides at the Gasserian ganglion into three branches [2,4]. The ophthalmic (V1) and maxillary (V2) divisions are purely sensory, supplying the upper face and midface, respectively; the mandibular division (V3) is mixed, carrying motor fibres to the muscles of mastication alongside sensory fibres to the lower face, teeth, and tongue. The proprioceptive neurons innervating craniofacial muscles are uniquely located in the mesencephalic trigeminal nucleus—the only site in the central nervous system where primary sensory neuronal cell bodies reside within the brain itself [2,4].

### 3.2. The Facial Nerve (CN VII)

The facial nerve exits the skull through the stylomastoid foramen, enters the parotid gland, and divides into five terminal branches—temporal, zygomatic, buccal, marginal mandibular, and cervical—supplying the muscles of facial expression, the stapedius, stylohyoid, and posterior belly of the digastric [2,5]. CN VII also carries taste fibres from the anterior two-thirds of the tongue (via the chorda tympani) and parasympathetic secretomotor fibres to the lacrimal, submandibular, and sublingual glands [3].

It is important to distinguish the voluntary somatic motor fibres to the muscles of expression, arising from the facial motor nucleus, from the secretomotor parasympathetic fibres: the greater petrosal nerve (lacrimal gland via pterygopalatine ganglion) and the chorda tympani (submandibular and sublingual glands via submandibular ganglion) arise from the superior salivatory nucleus and are autonomic, not voluntary. This distinction is clinically relevant throughout this review: injury or anaesthetic diffusion may produce motor, sensory, or autonomic effects depending on the fibre types conveyed, and these categories should not be conflated.

### 3.3. Proprioception Without Classical Muscle Spindles

**Figure 1:**
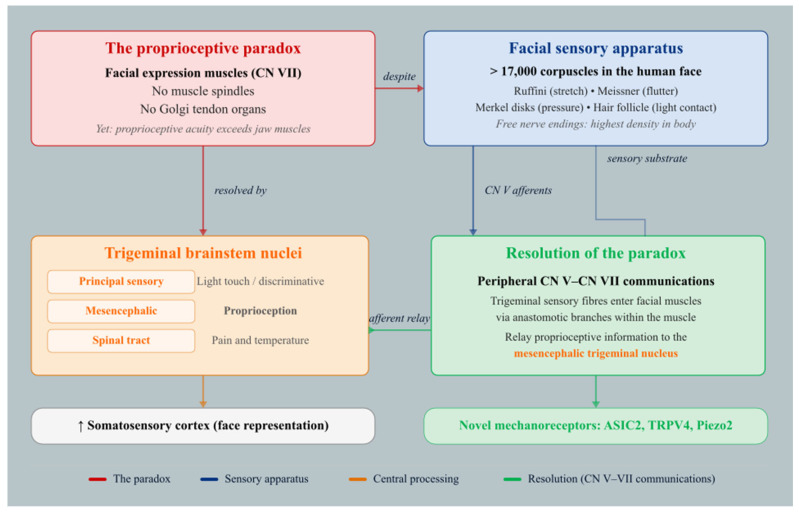
Trigeminal relay hypothesis for facial muscle proprioception.

The muscles of facial expression, controlled by CN VII, lack classical proprioceptive organs—muscle spindles and Golgi tendon organs—yet demonstrate proprioceptive acuity that, in the orofacial region, exceeds that of the spindle-bearing jaw muscles [2]. The facial sensory apparatus is substantial: over 17,000 corpuscles have been identified in the human face, served by Ruffini corpuscles (stretch), Meissner corpuscles (flutter), Merkel cell disks (pressure), and hair follicle receptors (light contact), with intraepidermal free nerve endings reaching their highest body-wide density in facial skin [22]. Somatosensory information travels via trigeminal axons to three brainstem subnuclei—the principal sensory (light touch), mesencephalic (proprioception), and spinal tract (pain and temperature) nuclei—before ascending to the face representation in the somatosensory cortex [22]. The resolution of this proprioceptive paradox (Figure 1) lies in the peripheral communications through which trigeminal sensory fibres gain access to facial muscles and relay proprioceptive information centrally.

## 4. Anatomical Mapping of Trigeminal–Facial Communications

### 4.1. Methodological Considerations

Three principal methods have mapped these communications, each with distinct advantages (see also the definitional controversy discussed below) (Table 1). Classical cadaveric dissection provides direct macroscopic visualisation but may underestimate fine connections; Sihler whole-mount staining reveals communications invisible to the naked eye; and immunohistochemistry confirms the mixed sensorimotor nature of fibres at the microscopic level. Iwai et al. [18] introduced a critical methodological advance by distinguishing genuine “nerve communication” (fibre exchange between trunks) from “nerve crossing” (epineurial contact without fibre interchange), substantially revising earlier prevalence estimates—particularly for V1, which showed 0% true communication under strict criteria despite 85.7% rates reported by Yang et al. [13] using standard Sihler methods.

Beyond methodological sensitivity, a fundamental definitional controversy pervades the literature. “Anastomosis” and “communication” have been applied inconsistently: Iwai et al. [19] require histologically confirmed fibre exchange, whereas earlier studies counted any topographic proximity or shared epineurial sheath [13]. This is not merely semantic. Nerves sharing an epineurium but maintaining separate perineuria—as at the forehead (supraorbital–temporal; Hwang et al., 2005) and chin (mental–marginal mandibular; Hwang et al., 2007)—may or may not exchange fibres [23,24]. Functionally, a trigeminal branch travelling alongside a facial motor branch within a shared epineurium still delivers proprioceptive signals to the shared territory. Surgically, any structure sharing a connective-tissue sheath with a nerve risks inadvertent division. Readers should note the methodological basis underpinning each prevalence figure: strict-criterion [12] figures are most conservative; Sihler [13] figures are intermediate; cadaveric pooled data [10] are the most inclusive.

### 4.2. Communications at the Facial Trunk and Major Divisions

Before the facial nerve divides, it receives communications from the auriculotemporal nerve (V3) and the great auricular nerve (C2–C3) deep within the parotid gland. Kwak et al. [8], in 30 Korean half-heads, provided detailed morphometric characterisation (Table 2): communicating auriculotemporal nerve branches arose near the ATN root and passed laterally to join the temporofacial division within the parotid substance. Namking et al. [7] described these branches as lying deep within the parotid, placing them at risk during parotidectomy. In 26.7% of specimens a minor facial nerve trunk was present alongside the main trunk, invariably entering the cervicofacial division [8].

### 4.3. Communications by Facial Region

**Figure 2:**
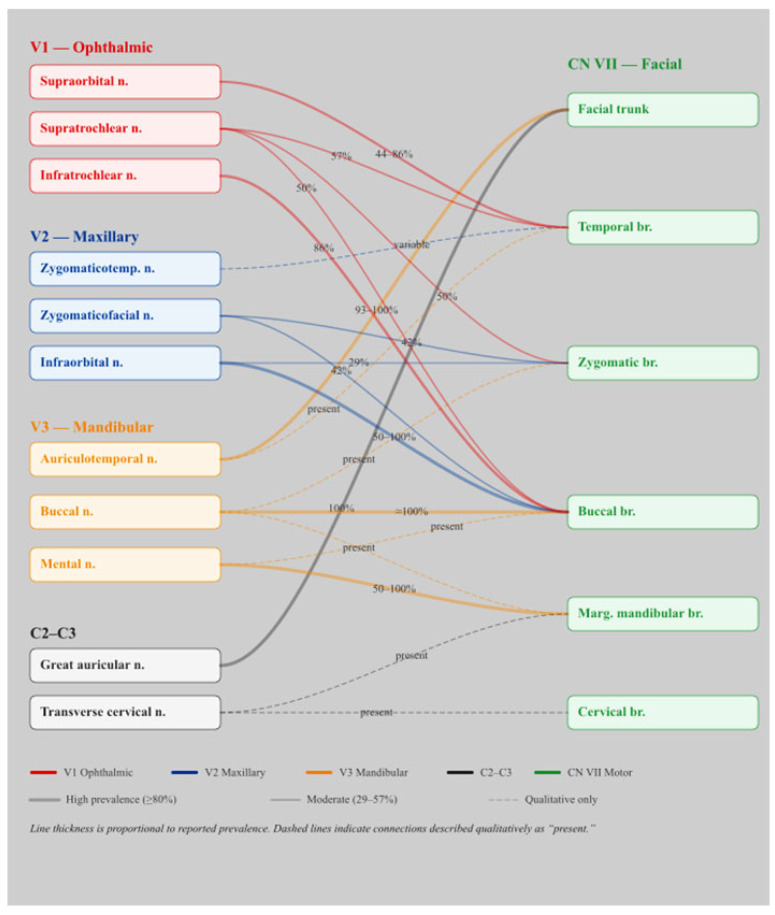
Schematic overview of the peripheral communications between the trigeminal (CN V) and facial (CN VII) nerves documented in Section 4.3. Left column: sensory branches grouped by trigeminal division—ophthalmic (V1, coral), maxillary (V2, blue), mandibular (V3, amber)—plus cervical plexus contributions (C2–C3, grey). Right column: motor branches of the facial nerve (teal), arranged from the undivided facial trunk superiorly to the cervical branch inferiorly. Each curved connector represents a documented anastomosis; line thick-ness is proportional to the reported prevalence, which is displayed as a numeric label along each curve. Solid lines denote connections for which quantitative prevalence data are available from cadaveric dissection or Sihler whole-mount staining studies; dashed lines indicate connections reported qualitatively as “present” without a specific percentage. Note that the buccal branch of CN VII receives the greatest number of trigeminal inputs (six), spanning all three divisions, whereas the facial trunk communications arise exclusively from the auriculotemporal nerve (V3, 93–100%) and the great auricular nerve (C2–C3, 100%). Prevalence figures are drawn from Kwak et al. [8], Hwang et al. [23,24], Yang et al. [13], Tansatit et al. [11], Iwanaga et al. [26], and Iwai et al. [18]; ranges reflect methodological differences between cadaveric dissection and Sihler staining techniques (see Section 3.1).

The facial–trigeminal communications by facial region are summarised in Figure 2.

#### 4.3.1. Temporal Branch

The temporal branch receives connections from the zygomaticotemporal nerve (V2), the horizontal branch of the supraorbital nerve (V1), and the auriculotemporal nerve (V3) (Table S1, Panels B and G; [2,23,27]). Yang et al. [13], using modified Sihler staining in 14 hemisectioned face specimens, provided a systematic atlas by dividing the periorbital region into four functional zones—superciliary (supraorbital–temporal anastomosis within orbicularis oculi), nasion (long ascending buccal branch twigs interconnecting with infratrochlear and supratrochlear nerves), and zygomatic (zygomaticofacial–zygomatic branch communication). Yang et al. [13] designated these collectively as the “superior facial nervous anastomosis” (SFNA) and noted that trauma or misdirected injection in these convergence zones could simultaneously produce both sensory and motor dysfunction.

#### 4.3.2. Zygomatic Branch

The zygomatic branch anastomoses with the buccal nerve (V3) and the zygomaticofacial nerve (V2), the latter potentially carrying postganglionic parasympathetic fibres to the lacrimal gland (Table S1, Panels B and C; [2,12]). Additional connections with the auriculotemporal, supraorbital, buccal, and supratrochlear nerves have been documented [2,13].

The zygomaticofacial–zygomatic branch connection carries postganglionic parasympathetic fibres from the pterygopalatine ganglion to the lacrimal gland via a communicating branch to the lacrimal nerve (V1). This is an autonomic (secretomotor), not a voluntary motor, pathway: its interruption during orbital or midface surgery risks ipsilateral dry eye rather than motor weakness—a clinically important distinction, since the communication runs alongside voluntary motor branches of CN VII.

#### 4.3.3. Buccal Branch

The buccal branch has the richest trigeminal connections. Kwak et al. [8] classified facial nerve branching into four types by buccal branch origin, finding buccal–zygomatic interconnections in 70% and buccal–marginal mandibular communications in 42% of specimens—frequencies notably higher than the 6.3% and 15% reported in Caucasian series [8,28], suggesting ethnic variation. Once the buccal branches exit the parotid, the buccal nerve of V3 communicates via the “communicating buccal nerve,” a thick anastomotic branch located at the outer layer of the buccinator deep fascia or within the buccinator and orbic-ularis oris muscles [2,12]. This connection has been implicated in spontaneous recovery of facial function after nerve transection [3,29]. The infraorbital nerve (V2) communicates with the buccal branch with near-universal prevalence, forming the infraorbital plexus—a sensorimotor convergence zone of approximately 36 mm diameter centred 22 mm below the infraorbital foramen [2,11,30].

#### 4.3.4. Marginal Mandibular Branch

The marginal mandibular branch receives a constant (100%) communication from the mental nerve (V3) [24,26]. Hwang et al. [24], dissecting 23 hemifaces, localised all connection points within a com-pact 6 × 12 mm rectangle centered between the lower vermilion border and gnathion, with a mean of 8.26 ± 2.49 sites per specimen. Histologically, the nerves shared a common epineurium but maintained separate perineuria (Table S1, Panel H)—a pattern that mirrors the forehead (common epineurium, separate perineuria) and contrasts with the midface, where both layers are shared [9,23]. Iwanaga et al. [26] identified two topographic types of this communication: a “superior type” ending in the lower lip and an “anterior type” extending to the chin, with a mean diameter of 0.4 mm and 1–8 communicating branches per specimen.

Won et al. [31], using Sihler staining, classified the mental nerve into four terminal branches and confirmed invariable communications with both the marginal mandibular branch (100%) and the cervical branch (100%) of CN VII. In addition, the buccal nerve (V3) anastomosed with the mental nerve in 60% of specimens—a bypass route that may explain the 15–20% failure rate of mandibular dental anaesthesia [31,32].

#### 4.3.5. Cervical Branch and Superficial Cervical Ansa

The cervical branch of CN VII communicates with the marginal mandibular branch in approximately 24.7% of cases [2,33,34]. The superficial cervical ansa—a loop between the cervical branch of CN VII and the transverse cervical nerve (C2–C3)—creates a mixed sensory–motor network across the lower face and upper neck (Table S1, Panel E; [35,36]).

### 4.4. Quantitative Summary by Trigeminal Division

Using the rigorous criteria of Iwai et al. [18], the mandibular division (V3) provides the most consistent communications (approaching universality), the maxillary division (V2) communicates in roughly half of specimens, and the ophthalmic division (V1) may contribute no true fibre exchanges at all (Table 1; Table S1, Panel G). These findings sug-gest that classical cadaveric literature likely overestimates V1 rates by conflating fibre exchange with topographic proximity.

Comprehensive regional mapping data, including prevalence, connective-tissue architecture, and key references for all twenty peripheral anastomoses, are provided in Table S1 (Supplementary Materials).

### 4.5. The Chorda Tympani–Lingual Nerve Junction

**Figure 3:**
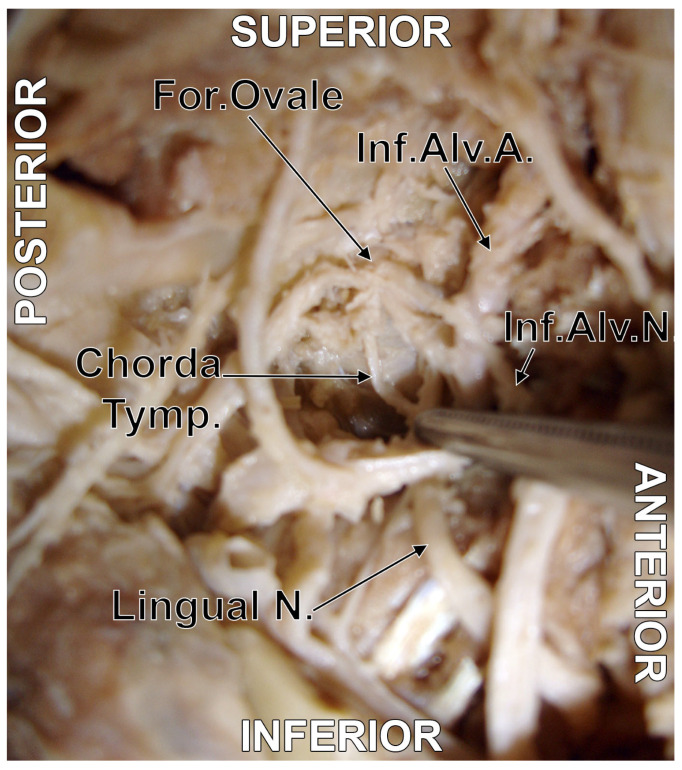
The chorda tympani–lingual nerve junction. Original dissection of the right infratemporal fossa. Infero-lateral view. Key labelled structures: chorda tympani, lingual nerve (V3), junction point, lateral pterygoid muscle.

An important deep trigeminal–facial connection occurs at the junction of the chorda tympani (CN VII) and the lingual nerve (V3) (Figure 3). The chorda tympani arises within the facial canal, traverses the middle ear, exits through the petrotympanic fissure, and joins the lingual nerve deep to the lateral pterygoid muscle [3,36]. This junction is established at the 18 mm embryonic stage, well before most superficial communications, consistent with classical descriptions of facial musculature development [3,37,38]. Through it, the chorda tympani contributes gustatory fibres for the anterior two-thirds of the tongue and parasympathetic fibres to the submandibular ganglion [3,36,39].

Clinically, lingual nerve injury during lower third molar extraction simultaneously disrupts both V3 somatosensory fibres and chorda tympani fibres, causing hypoaesthesia and taste loss [40]. In face transplantation, coaptation of the lingual nerve can restore somatosensory feedback, while chorda tympani co-coaptation may restore taste to the anterior tongue [41,42]. Diamond et al. [3] further noted that the chorda tympani connects to the otic ganglion [43] and participates in additional communications with the tympanic plexus (CN IX), helping to explain the protean manifestations of Ram-say Hunt syndrome.

## 5. Histological Evidence of Sensorimotor Fibre Interchange

### 5.1. Immunohistochemical Proof

Beyond macroscopic mapping, immunohistochemical analysis has provided histological evidence of fibre exchange. The hallmarks—multiple perineuria within a common epineurium, and a VAChT-positive (motor) to VAChT-negative (sensory) gradient across the anastomotic zone—are consistently observed (Table S1, Panel H; [2,14]. Mohanty et al. [14] demonstrated pronounced intramuscular coupling at the buccinator, documenting a gradual shift from motor-predominant to sensory-predominant fibre composition when sampling from CN VII territory toward CN V territory—confirming directional fibre interchange. These findings validate that trigeminal sensory fibres genuinely enter facial muscles via CN VII branches and that the distal facial nerve is functionally a mixed nerve [4].

### 5.2. Axonal Mapping

Tereshenko et al. [16], using multi-marker immunofluorescence in human organ donors, found that the extracranial facial nerve trunk contained 11,778 ± 1138 total axons, of which only 77.7 ± 2.3% were cholinergic—roughly twice the earlier conventional counts [44,45,46]. Branch-level composition varied: temporal 3334 ± 2518 (67.9% motor); zygomatic 2985 ± 1269 (61.3%); buccal 6092 ± 1672 (53.0%); mandibular 2121 ± 594 (61.8%); cervical 1910 ± 229 (43.2%). The buccal branch carried the highest absolute number of non-cholinergic axons (2892 ± 1305), consistent with its extensive trigeminal communications. Critically, the distal branches contained a myelinated afferent population absent from the main trunk—evidence that afferent fibres enter peripherally through the trigeminal–facial communications [16]. All non-myelinated, non-cholinergic axons were TH-positive (sympathetic), ranging from 15% (mandibular) to 30% (buccal), and were hypothesised to modulate facial muscle tone independently of cortical control [16].

## 6. Proprioception in the Facial Muscles

### 6.1. The Trigeminal Relay Hypothesis

Baumel’s (1974) proposal that proprioceptive impulses from facial muscles travel via CN V branches remains the prevailing framework [6]. Tereshenko et al. [16] substantially refined this hypothesis using immunohistochemistry, retrograde tracing, whole-mount imaging, and electrophysiology in a selectively deafferented rat model. The human facial nerve was shown to contain only 85% cholinergic axons, with 15% non-cholinergic fibres; in the rat, 93.9% were motor, 4.4% CGRP-positive (afferent), and 2.4% TH-positive (sympathetic). CGRP immunoreactivity marks peptidergic nociceptive afferents rather than proprioceptive fibres per se; these fibres are therefore best characterised as afferent (primarily nociceptive) rather than proprioceptive. It should further be noted that cholinergic markers are not exclusively motor: cholinergic fibres have been identified in certain afferent systems, including vagal sensory pathways [47], so VAChT positivity alone cannot be treated as unambiguous evidence of motor fibre identity in mixed-nerve anastomoses. Retrograde tracing confirmed three central origins: facial nucleus motor neurons (4837 ± 227 cells), geniculate ganglion afferents (194 ± 50), and superior cervical ganglion sympathetics (121 ± 34). No labelled neurons were found in the trigeminal ganglion [16].

After cutaneous trigeminal deafferentation, whole-mount staining of facial muscles revealed abundant TH-positive (sympathetic) fibres terminating in muscle substance and along intramuscular vessels, but CGRP-positive fibres did not terminate in recognisable sensory endings within the muscle. Electrophysiological recordings demonstrated that touch and vibration stimuli on the skin evoked clear afferent nerve action potentials through the facial nerve (maximal RMS amplitudes up to 3.87 ± 2.51 µV), whereas muscle stretching produced no measurable proprioceptive response (0.12 ± 0.02 µV, indistinguishable from noise) [16]. The facial nerve thus conveys cutaneous mechanoreceptive but not intramuscular proprioceptive signals, and sympathetic fibres travel via the facial nerve to reach facial muscles.

### 6.2. Alternative Proprioceptive Mechanisms

Cutaneous mechanoreceptors activated by skin deformation during facial movements provide substantial proprioceptive feedback: the over 17,000 corpuscles documented in the human face encompass four functionally complementary receptor types collectively served by Aβ fibres with receptive fields of approximately 7–8 mm^2^ [2,22]. In addition, Cobo et al. [4] identified non-spindle proprioceptor-like structures within human facial muscles: capsulated and non-capsulated corpuscles displaying immunoreactivity for ASIC2, TRPV4, and Piezo2, with the highest density in the buccinator and orbicularis oris. These novel proprioceptors, confirmed by double immunofluorescence and confocal microscopy, support a genuine proprioceptive role in spindle-free muscles [4].

## 7. Clinical Significance

### 7.1. Facial Nerve Injury and Spontaneous Recovery

Martin and Helsper (1957, cited in Diamond et al., 2011) observed that some patients regained voluntary facial movement after surgical section of the facial nerve trunk, proposing that motor impulses reached the muscles via trigeminal fibres travelling along communicating branches [3,29]. DeLacure et al. [48] and Cheney et al. [49] specifically implicated the communicating buccal nerve as the responsible pathway. Mohanty et al. [14] extended this by demonstrating that the buccinator anastomotic zone contains both sensory and motor fibres in intimate proximity—an arrangement that could facilitate cross-reinnervation.

### 7.2. Facial Reanimation Surgery

The masseteric nerve (V3) has become the most commonly used donor for facial reanimation. Urban et al. [50], in a meta-analysis of 71 studies (1,532 patients), found that masseteric transfer produced higher Sunnybrook scores (47.7 ± 7.4 vs. 33.0 ± 6.4) and faster first movement (4.6 ± 2.6 vs. 6.3 ± 1.3 months) than hypoglossal transfer, while the lat-ter yielded better resting symmetry. Liu et al. [51] reported selective trigeminal motor branching transfer using the masseteric, deep temporal, and medial pterygoid nerves for different target muscles, with 100% eye closure recovery and significant improvement in oral commissure movement (*p* < 0.001). López Granados et al. [52] validated dual nerve transfer (hypoglossal + masseteric), with initial movement at three to four months and spontaneous movement by six months. Other options include cross-face nerve grafting, which uniquely enables emotion-driven smiling but requires a lengthy regeneration period; systematic reviews and meta-analyses of reanimation outcomes pro-vide comparative effectiveness data across these techniques [5,53,54].

### 7.3. Face Transplantation

Cobo et al. [2,4] emphasised that disruption of trigeminal–facial communications during tissue harvesting impairs proprioceptive capacity of transplanted facial muscles. Siemionow et al. [22], reviewing the first 11 reported cases, noted that the facial nerve was repaired in three of four patients with long-term follow-up but sensory nerves satisfactorily in only one, an asymmetry attributed to historically inadequate attention to sensory reconstruction. The challenges include high-positioned bony foramina, long canals, and short extraosseous nerve trunks; Siemionow et al. [22] advocated osteotomy of the supra-orbital, infraorbital, and mandibular canals to lengthen nerve stumps for coaptation.

Cortical reorganisation accompanying deafferentation is a further consideration: Farné et al. [55] showed that somatosensory perception of the transplanted hand was hampered during simultaneous facial stimulation, suggesting face–hand cortical competition during recovery [56]. Siemionow et al. [22] also provided normative two-point discrim-ination values (lateral forehead 13.4–15.0 mm; upper lip 3.25–7.66 mm; vermilion 2.4–4.6 mm) as benchmarks for monitoring sensory recovery.

### 7.4. Parotid and Midface Surgery

The communicating auriculotemporal branches deep within the parotid are at risk during parotidectomy [7,8]. The infraorbital plexus—centred 22 mm below the infraorbital foramen—represents a danger zone in midface surgery, filler injections, and Le Fort osteotomies [11].

### 7.5. Botulinum Toxin

The trigeminal–facial communications provide pathways for unintended spread of botulinum toxin beyond the targeted territory. Chemodenervation of the orbicularis oculi could theoretically affect buccinator or zygomaticus function via the shared plexus [2].

### 7.6. Dental Anaesthesia and the Sensorimotor Plexuses

#### 7.6.1. The Infraorbital Plexus

The three most clinically encountered trigeminal nerve blocks in the midface and lower face—infraorbital, mental/incisive, and buccal—are conventionally taught as purely sensory techniques [57]. Viewed through the lens of the sensorimotor plexuses, their effects extend to motor pathways, proprioceptive feedback, and adjacent nerve distributions.

**Figure 4:**
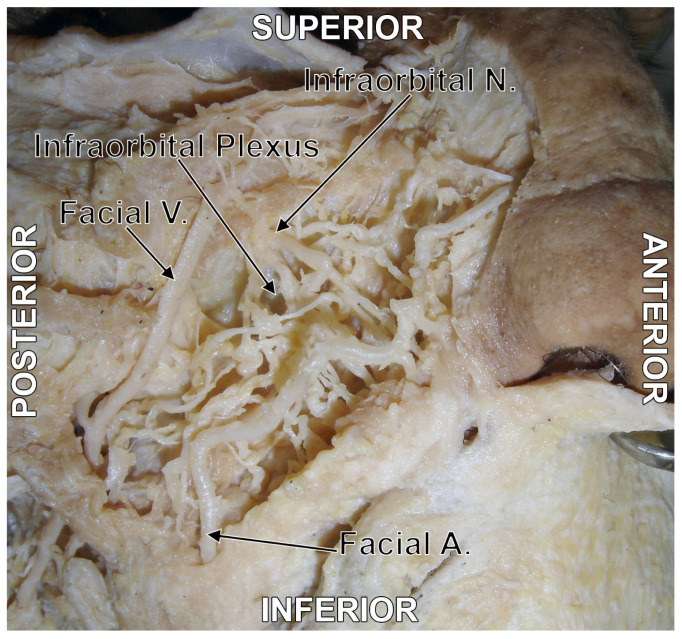
Original dissection of the infraorbital plexus. Right side. Infero-lateral view. Key labelled structures: infraorbital nerve (V2), buccal branch of CN VII, infraorbital plexus convergence zone.

The infraorbital plexus (Figure 4), the dense sensorimotor convergence zone located within a circle of approximately 36 mm diameter centred 22 mm inferior to the infraorbital foramen [2,30], constitutes a critical surgical and anaesthetic landmark in the midface. Within this zone, branches of the infraorbital nerve (V2) and the buccal branch of CN VII intertwine in up to 100% of Sihler-stained specimens [13], while Tansatit et al. [11] documented three distinct communication patterns (triple, double, and single) between the buccal trunk and the lateral labial branch of the infraorbital nerve, deep to the levator labii superioris. Disruption of these communications during Le Fort osteotomies, mid-face fracture repair, filler injections, or infraorbital nerve blocks can simultaneously compromise both sensory and motor function in the perioral region.

#### 7.6.2. Accessory Infraorbital Foramina and Clinical Anatomy

The clinical topography of this plexus is further complicated by the variable osseous anatomy of the infraorbital foramen itself. In a retrospective cone beam computed tomography (CBCT) study of 200 patients (400 maxillae), Rusu et al. [21] demonstrated that accessory infraorbital foramina are more common than previously appreciated when assessed with cross-sectional imaging [58,59], and, critically, that two morphologically distinct categories must be distinguished. True accessory infraorbital foramina (AIOF), located inferior to the infraorbital margin and connected by canaliculi deriving from the infra-orbital canal, were found in 31 of 400 maxillae (7.8%); of these, 94% were single, 3% double, and in one exceptional case three accessory foraminules transformed the anterior antral wall into a veritable lamina cribriformis infraorbitalis. A separate category, accessory foramina located within the sutura notha—the constant vascular groove in front of the anterior lacrimal crest first described by Macalister (1884)—were connected to the infraorbital canal (AIOF(SN)) in 15 maxillae (3.8%); however, all maxillae negative for AIOF(SN) still displayed the sutura notha and its associated vascular foramina of Macalister, which receive branches from the extraosseous infraorbital artery rather than from the infraorbital canal [21]. This distinction is important because earlier dry-bone studies, which constituted the bulk of the prior literature, could not reliably differentiate true accessory foramina (carrying neural or neurovascular branches from the infraorbital canal) from the vascular foramina of Macalister (carrying arterial twigs entering the bone from outside). Indeed, of more than twenty published studies on accessory infraorbital foramina conducted on dry skulls, only Zhang et al. [60] had previously attempted this distinction, and their transillumination method found no connection between the sutura notha foramina and the infraorbital canal, a conclusion that the CBCT evidence of Rusu et al. [21] partially contradicts, having demonstrated IOC-derived canaliculi supplying sutura notha foramina in 6% of cases.

The significance of these findings for the trigeminal–facial infraorbital plexus is twofold. First, accessory infraorbital foramina represent additional exit points through which branches of the infraorbital nerve—and, by extension, their communications with CN VII—emerge onto the anterior maxillary surface. A standard infraorbital nerve block targeting the main foramen may therefore fail to anaesthetise branches exiting through accessory foramina located several millimetres away, contributing to incomplete anaesthesia of the upper lip and midface. Second, the sutura notha, even when its foramina are purely vascular, marks a site at risk for haemorrhage during local surgical approaches to the anterior maxilla and medial orbit; when its foramina are true AIOF(SN), the risk extends to inadvertent neural injury. CBCT assessment of the infraorbital region prior to midface surgery, implant placement, or aesthetic procedures is therefore recommended to map both the main and accessory foramina and to characterise their relationship to the infraorbital canal [21].

#### 7.6.3. The Labiomental Plexus

**Figure 5:**
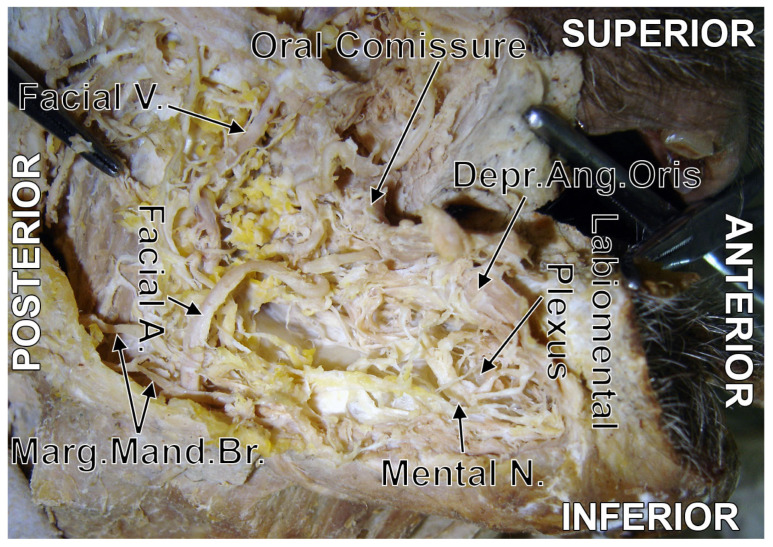
Original dissection of the labiomental plexus. Right side. Lateral view. Key labelled structures: mental nerve (V3), marginal mandibular branch of CN VII, communicating branches.

The labiomental plexus. The mental nerve branches fan out from the mental foramen into the labiomental plexus (Figure 5), where they anastomose with the marginal mandibular (100%) and cervical (100%) branches of CN VII [24,26,31]. A mental nerve block, therefore, produces not only sensory loss but transient lower-lip depressor weakness [26]. Won et al. [31] demonstrated that the buccal nerve anastomoses with the mental nerve in 60% of cases, creating a bypass that may cause mental block failure for the lateral lower lip.

#### 7.6.4. The Buccal Plexus

**Figure 6:**
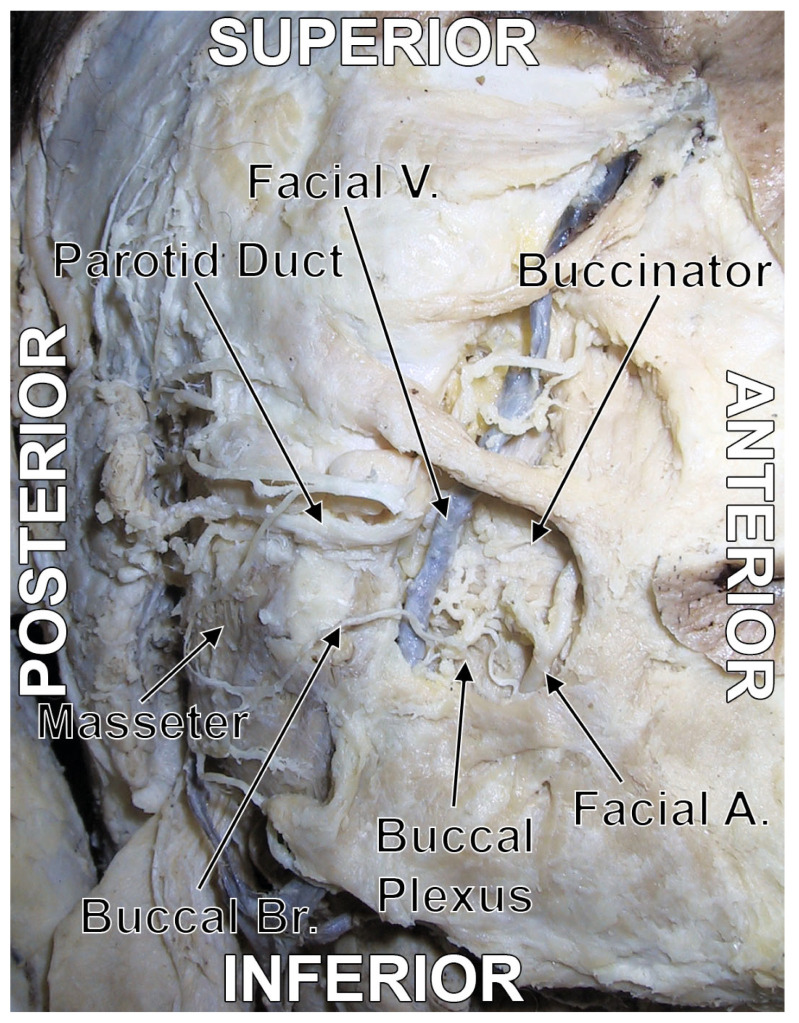
Original dissection of the buccal plexus. Right side. Antero-lateral view. Key labelled structures: buccal branch of CN VII, buccal nerve (V3), communicating branches.

The buccal plexus. The long buccal nerve (V3) forms the buccal plexus (Figure 6) at the outer surface of the buccinator, where it interweaves with the buccal branches of CN VII [13,14]. Blocking the buccal nerve can produce unexpected numbness of both lips via anastomotic connections with the infraorbital nerve superiorly and the mental nerve inferiorly [3].

#### 7.6.5. Clinical Evidence, Anaesthetic Diffusion, and Sensorimotor Disruption

Clinical evidence. Sülek et al. (2025 [61]) found identical success rates of 70% for mental/incisive block and IAN block for mandibular premolars with irreversible pulpitis, with better first-premolar efficacy (76.9% vs. 64.7%) attributable to variable buccal nerve contributions. Rujirawan et al. [62], in a network meta-analysis of 28 RCTs, confirmed that combined multi-nerve strategies significantly outperform IAN block alone (RR 2.02 and 1.86). He and Zhang [63] validated a 3D-printed indicator-guided combined IAN/lingual/buccal block with significantly fewer motor complications (1.9% vs. 8.6%, *p* = 0.030). Moodley et al. [64], reviewing 149 studies, concluded that block efficacy (70–98%) depends critically on landmark identification, accessory foramina recognition, and understanding of neural interconnections.

Anaesthetic spread and neurotoxicity. The communications serve as conduits for anaesthetic diffusion between nerve territories, explaining phenomena such as lower lip paralysis after mental nerve block [26] and facial paralysis following intraoral injection (12 of 18 cases attributed to diffusion via communicating branches [65]). In the TMJ region, Chęciński et al. [66] found transient facial paralysis in 14–65% of patients following intra-articular injections, attributed to diffusion through auriculotemporal–facial communications. Verlinde et al. [67] identified three neurotoxicity pathways (intrinsic caspase, PI3K, MAPK) particularly relevant to the small-calibre communicating branches with thin perineurial barriers.

Disruption of sensorimotor loops. Pilurzi et al. [68] demonstrated that cutaneous trigeminal afferents produce short-latency afferent inhibition (SAI) of face primary motor cortex at 15–30 ms, while facial nerve stimulation produces long-latency afferent inhibition (LAI) at 100–200 ms, with only the latter capable of inducing LTP-like plasticity. Trigeminal anaesthesia eliminates the rapid SAI pathway, which may explain the disproportionate facial clumsiness patients report after extensive dental blocks.

### 7.7. Perineural Tumour Spread

Perineural tumour spread (PNTS) occurs most commonly along the trigeminal and facial nerves [15]. The pre-existing neural communications—particularly the Vidian nerve, greater superficial petrosal nerve, and auriculotemporal nerve—provide conduits through which PNTS can jump between cranial nerves. Up to 40% of patients with PNTS may be asymptomatic [15]. Key imaging signs include fat-pad obliteration at neural foramina, nerve thickening and enhancement on MRI, and denervation muscle changes.

Schmalfuss et al. [69], in 15 patients with auriculotemporal nerve PNTS, found definitive imaging signs in 14, with treatment delays averaging 8.7 months when the nerve was not systematically evaluated. Tong et al. [70] distinguished perineural invasion (PNI, histological finding) from perineural spread (PNS, clinically detectable, worse prognosis), and identified the same periocular trigeminal–facial communications documented by Yang et al. [13] as clinically significant tumour routes. The zonal classification of Panizza et al. [71] categorises PNS by extent: Zone 1 (five-year survival 84%), Zone 2 (64%), and Zone 3 (15%, generally unresectable).

### 7.8. Hemifacial Spasm, Synkinesis, and Ramsay Hunt Syndrome

Diamond et al. [3] cited reports that facial nerve block for hemifacial spasm occasionally produces nystagmus, which is attributed to vestibular–facial communication. The trigeminal–facial communications may also explain variable patterns of synkinesis after aberrant facial nerve regeneration. Ramsay Hunt syndrome, caused by varicella–zoster reactivation in the geniculate ganglion, can affect multiple cranial nerves (V, VII, IX, XI, XII) in unpredictable combinations; the communicating branches provide the anatomical explanation for this variability [3,4].

### 7.9. Trigeminal Neuropathy as a Sentinel Sign

Isolated trigeminal neuropathy, particularly the “numb chin syndrome,” carries a well-documented association with distant metastatic disease or local jaw malignancy. Because of the dense mental nerve–marginal mandibular communications, patients may also exhibit subtle motor findings that confuse the clinical picture [72].

## 8. Central Trigeminal–Facial Circuits

The brainstem reflex arcs provide essential context: the blink reflex (V1 → trigeminal spinal nucleus → reticular formation → facial motor nucleus → orbicularis oculi), the jaw-opening reflex, and the salivary reflex illustrate intimate central integration of CN V and CN VII [73,74].

## 9. Communications Beyond the Trigeminal–Facial Axis

### 9.1. Facial–Glossopharyngeal Communications (CN VII–IX)

**Figure 7:**
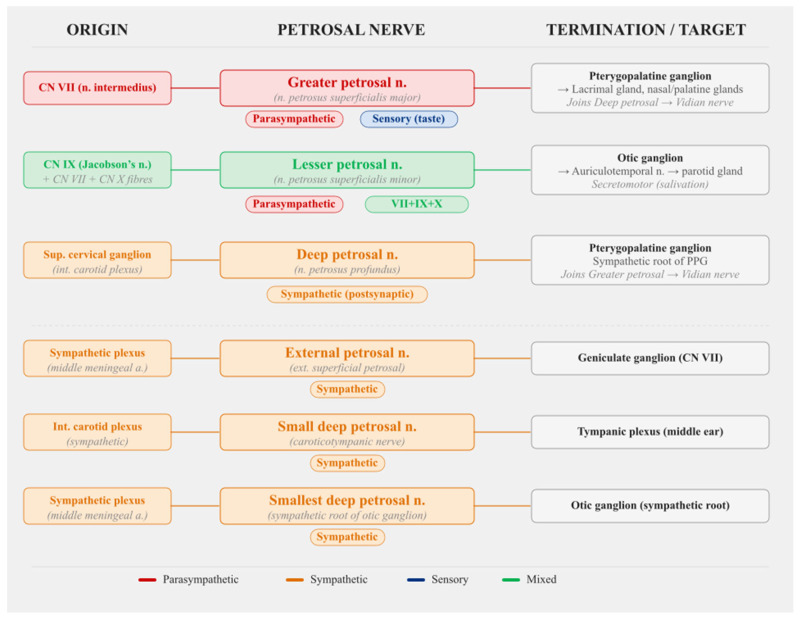
Schematic overview of the six petrosal nerves as classified by Tubbs et al. [81], the only comprehensive review in the modern anatomical literature to systematise the nomenclature for all six structures under a single framework. Each row depicts one petrosal nerve, showing its origin (**left**), course designation with alternative nomenclature in parentheses (**centre**), and termination or target ganglion (**right**). Colour-coded badges indicate the fibre type carried by each nerve: parasympathetic (red) or sympathetic (orange); the greater petrosal nerve additionally carries sensory fibres subserving taste from the soft palate (dark blue badge). The greater petrosal nerve (CN VII, nervus intermedius [82]) and the deep petrosal nerve (superior cervical ganglion, via the internal carotid plexus) merge at the foramen lacerum to form the Vidian nerve (nerve of the pterygoid canal), which conveys both parasympathetic and sympathetic fibres to the pterygopalatine ganglion—this convergence is annotated within the termination boxes of rows 1 and 3. The lesser petrosal nerve is classified as parasympathetic; although it receives contributing fibres from the facial nerve (nervus intermedius) and the vagus nerve (Arnold’s nerve) in addition to its principal glossopharyngeal origin (Jacobson’s nerve via the tympanic plexus), these contributions are autonomic in nature and do not alter the nerve’s fundamentally parasympathetic function. The four sympathetic petrosal nerves—external, deep, small deep, and smallest deep—are grouped below the dashed separator; all derive from the sympathetic plexuses surrounding either the internal carotid artery or the middle meningeal artery. Of these, the external petrosal nerve is regarded as inconstant by some authors.

The most documented CN VII–IX connections occur at the level of the temporal bone [75]. The lesser petrosal nerve receives a communicating branch at the geniculate ganglion, with Kakizawa et al. [3,76] identifying three distinct junction patterns. The facial nerve also sends a twig to the tympanic plexus (Jacobson’s nerve). Peripherally, the ansa of von Haller—a direct CN VII–IX communicating loop arising after the stylomastoid foramen—was found in 4% of sides by Salame et al. [77,78], while Kawai et al. [3,79] identified three types of facial–glossopharyngeal communication at the posterior digastric region in 18.8% of 490 half-heads. Müller and Rude [80] described a separate skull-base anastomosis transmitting parasympathetic and gustatory fibres, and the geniculate ganglion receives the external petrosal nerve from the sympathetic plexus around the middle meningeal artery [78]. The petrosal nerve communications are summarised in Figure 7.

### 9.2. Facial–Vagal Communications (CN VII–X) and Arnold’s Nerve

Arnold’s nerve, the auricular branch of the vagus, shares embryo-logical origins with the facial nerve [3,38] and innervates the concha and external acoustic meatus, producing the Arnold’s cough reflex—present in approximately 25% of adults with chronic cough [83]. The convergence of Arnold’s nerve fibres at the geniculate ganglion creates a three-nerve junction (CN VII–IX–X) that helps explain the diverse manifestations of Ramsay Hunt syndrome [3].

### 9.3. Trigeminal–Glossopharyngeal (CN V–IX) and Trigeminal–Vagal (CN V–X) Connections

Constant intralingual anastomoses between the lingual nerve (V3), CN IX, and CN XII have been demonstrated by Touré et al. [84] and Păduraru and Rusu [20]. These explain preserved taste after chorda tympani section through anterior overextension of glossopharyngeal lingual branches [5,85]. Direct CN V–X connections are rare; their in-teraction is primarily indirect, but functional convergence occurs at the external ear, where the auriculotemporal nerve (V3), Arnold’s nerve (CN X), and great auricular nerve (C2–C3) create overlapping sensory territories that explain referred otalgia [78].

### 9.4. Intra-Trigeminal Anastomoses

Intra-trigeminal anastomoses are reviewed here because they form the anatomical scaffolding within which CN V–CN VII communications operate: V3 connections that share common trunks can carry trigeminal fibres into facial nerve territories by routes not immediately apparent from primary CN V–CN VII communications, and have direct relevance to perineural tumour spread pathways, anaesthetic diffusion routes, and the clinical implications discussed in Section 7.

**Figure 8:**
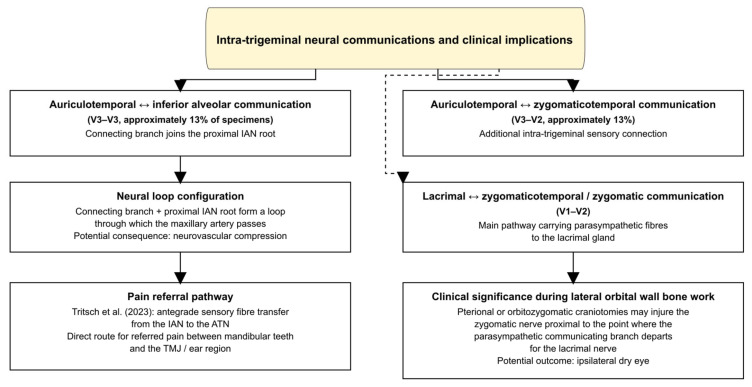
Intra-trigeminal anastomoses.

The auriculotemporal–inferior alveolar nerve communication (V3–V3) was found in approximately 13% of specimens [86], with the connecting branch and proximal IAN root forming a neural loop through which the maxillary artery passes—potentially causing neurovascular compression and referred pain [87]. Tritsch et al. [88] showed antegrade sensory fibre transfer from the IAN to the ATN; variant branches of the IAN further diversify this region’s anatomy [89], providing a direct pathway for pain referral between mandibular teeth and the TMJ/ear region. Additional intra-trigeminal connections include the auriculotemporal–zygomaticotemporal communication (V3–V2, ~13%) and the lacrimal–zygomaticotemporal/zygomatic communication (V1–V2), the main pathway for parasympathetic fibres to the lacrimal gland, clinically significant because bone work at the lateral orbital wall during pterional or orbitozygomatic craniotomies may injure the zygomatic nerve proximal to the point at which the parasympathetic communicating branch departs for the lacrimal nerve, potentially resulting in ipsilateral dry eye [78]. The full map of intra-trigeminal communications is illustrated in Figure 8.

## 10. Future Directions

Several frontiers remain. The identification of ASIC2, TRPV4, and Piezo2 in novel facial muscle proprioceptors [4] opens the door to mechanistic studies of mechanotransduction in spindle-free muscles. The substantial sympathetic axonal population in the facial nerve [16] raises questions about autonomic contributions to muscle atrophy in facial palsy and to postoperative synkinesis. The differential contributions of cutaneous (SAI) and proprioceptive (LAI) pathways to motor cortex excitability [68] suggest that targeted sensory rehabilitation protocols could enhance motor recovery after facial nerve injury. In imaging, three-dimensional high-resolution ultrasound and magnetic resonance microscopy hold promise for preoperative mapping of trigeminal–facial communications. In bioengineering, nerve guidance conduits loaded with growth factors or stem cells, and neuroprosthetic devices controlled by electromyographic signals, represent emerging approaches to facial nerve repair that could exploit the trigeminal–facial communications as biological scaffolds or alternative innervation pathways. The animal model developed by Tereshenko et al. [90]—permitting selective deafferentation or deefferentation of the face in rats—provides an experimental platform for testing these interventions. The quantitative axonal data from Tereshenko et al. [16] now establish precise motor axon count benchmarks for the facial, hypoglossal, and masseteric nerves [91], which should inform the design of nerve transfer protocols by providing evidence-based targets for axon count matching between donor and recipient nerves. The validation of dual nerve transfer [53] and selective trigeminal motor branching transfer [51] represents an advance toward increasingly targeted reanimation protocols exploiting the anatomical trigeminal–facial connections.

## 11. Conclusions

The trigeminal and facial nerves are not isolated sensory and motor pathways but intimately interconnected partners whose peripheral communications form sensorimotor plexuses throughout the face. The axonal cartography of Tereshenko et al. [16] has fundamentally revised the classical view of the extracranial facial nerve, revealing it as a mixed nerve containing motor, sympathetic, and afferent components, with the distal branches acquiring additional myelinated afferents through the trigeminal–facial communications. Won et al. [31] demonstrated invariable mental nerve–facial nerve anastomoses alongside a buccal nerve bypass present in 60% of specimens, clarifying the anatomical basis for dental anaesthesia failure. These connections are clinically consequential across multiple disciplines, from spontaneous recovery after nerve injury and nerve transfer planning to face transplantation, from perineural tumour spread along the auriculotemporal nerve [69] to the unexpected effects of local anaesthesia diffusing through communicating branches. Surgeons, radiologists, neurologists, dentists, and aesthetic practitioners benefit from understanding this network, and ongoing research in molecular mechanotransduction, high-resolution imaging, and nerve bioengineering promises to expand that understanding further.

## Figures and Tables

**Table 1:** Comparison of Methods Used to Map Trigeminal–Facial Communications. Prevalence data are reported as overall communication rates by trigeminal division. Abbreviations: V1 = ophthalmic; V2 = maxillary; V3 = mandibular; VAChT = vesicular acetylcholine transporter.

Method	Principle	V1 %	V2 %	V3 %	Limitations/Key Insight	Key References
Cadaveric dissection	Macroscopic visualisation under magnification	33.8% ± 19.5%	95.0% ± 8.0%	76.7% ± 38.5%	May underestimate fine connections; pooled data show high variability	[10] (pooled); [7,8,11,12]
Sihler whole-mount staining	Renders tissue translucent; stains myelinated fibres	85.7% (Yang); 0% strict (Iwai)	100% (Yang); 50% strict (Iwai)	100% (both Yang and Iwai); 50% via mental n. (Iwai strict)	Reveals connections invisible to the naked eye; strict criteria distinguish true fibre exchange from epineurial crossing	[13] (14 hemifaces); [18] (6 whole faces)
Immunohistochemistry	Molecular markers (VAChT, TH) identify motor and sympathetic fibres respectively; Masson trichrome reveals connective-tissue architecture (separate vs. shared perineurium) but is a structural stain, not an immunohistochemical marker	N/A	N/A	N/A	Confirms mixed sensorimotor nature; VAChT gradient shows gradual sensory-to-motor transition; two or more perineuria in a single epineurium	[2,14]

Notes. Cadaveric dissection figures pooled from Hwang et al. [10] (individual study n: 10–30 hemifaces). Yang et al. [13] Sihler staining: 14 hemisectioned adult faces. Iwai et al. [18] strict-criterion Sihler: 6 whole adult faces (12 hemifaces). The V1 discrepancy (85.7% Yang vs. 0% Iwai strict) reflects the definitional distinction between epineurial contact without fibre exchange (“nerve crossing”) and confirmed fibre interchange (“nerve communication”); V1 branches show topographic proximity to CN VII but perineurial fibre exchange has not been confirmed under strict criteria. Immunohistochemistry column refers to the buccinator anastomotic zone only [14].

**Table 2:** Morphometric and Branching Data for the Facial Nerve Trunk and Its Intraparotid Communications. All data from Kwak et al. [8], 30 Korean half-heads, unless otherwise indicated. Abbreviations: CATN = communicating auriculotemporal nerve; ATN = auriculotemporal nerve.

Parameter	Value	Source
Skin surface to facial nerve trunk depth	21.0 ± 3.1 mm (range 16.9–28.8)	[8]
Stylomastoid foramen to furcation distance	13.0 ± 2.8 mm (range 8.8–16.4)	[8]
Bifurcation pattern (temporo-/cervicofacial)	86.7%	[8]
Trifurcation pattern	13.3%	[8]
Minor facial trunk present	26.7% (all entered cervicofacial division)	[8]
ATN–facial trunk communication prevalence	93.3% (cadaveric); 100% (Sihler)	[8,12]
CATN branch number per specimen	2–4; three-branch pattern most common (46.7%)	[8]
Single CATN branch	Never observed (0%)	[8]
Great auricular n.–facial trunk communication	100%	[2,25]

## Data Availability

No new data were created or analysed in this study. Data sharing is not applicable to this article.

## Supplementary Materials

The following supporting information can be downloaded at: https://www.mdpi.com/article/10.3390/diagnostics16121855/s1. Table S1: Comprehensive Anatomical Map of Peripheral Trigeminal–Facial Nerve Communications. The table is organised into eight panels: Panel A (trunk-level and intraparotid communications); Panel B (upper face: forehead and periorbital region); Panel C (midface: zygomatic, buccal, and infraorbital region); Panel D (lower face: mandibular and mental region); Panel E (cervical region); Panel F (deep intracranial and infratemporal communications); Panel G (summary of prevalence by trigeminal division and method); and Panel H (connective-tissue architecture by facial region). For each communication, the table reports the trigeminal or cervical branch of origin, the facial nerve target, cadaveric and Sihler-staining prevalence, the number of communicating branches, connective-tissue architecture, topographic location, target muscles, and key references.

## Abbreviations

The following abbreviations are used in this manuscript:

AIOF	accessory infraorbital foramen/foramina
AIOF(SN)	accessory infraorbital foramen in sutura notha
ASIC2	acid-sensing ion channel 2
ATN	auriculotemporal nerve
CATN	communicating auriculotemporal nerve
CBCT	cone beam computed tomography
CGRP	calcitonin gene-related peptide
CN V	trigeminal nerve
CN VII	facial nerve
CN IX	glossopharyngeal nerve
CN X	vagus nerve
CN XI	accessory nerve
CN XII	hypoglossal nerve
IAN	inferior alveolar nerve
ION	infraorbital nerve
LAI	long-latency afferent inhibition
LLSAN	levator labii superioris alaeque nasi
LTP	long-term potentiation
MAPK	mitogen-activated protein kinase
MRI	magnetic resonance imaging
PI3K	phosphoinositide 3-kinase
Piezo2	piezo type mechanosensitive ion channel component 2
PNI	perineural invasion
PNS	perineural spread
PNTS	perineural tumour spread
RCT	randomised controlled trial
RMS	root mean square
RR	risk ratio
SAI	short-latency afferent inhibition
SANRA	Scale for the Assessment of Narrative Review Articles
SFNA	superior facial nervous anastomosis
TH	tyrosine hydroxylase
TMJ	temporomandibular joint
TRPV4	transient receptor potential vanilloid 4
V1	ophthalmic division of the trigeminal nerve
V2	maxillary division of the trigeminal nerve
V3	mandibular division of the trigeminal nerve
VAChT	vesicular acetylcholine transporter
