# Supplementary material for: Trigeminal–Facial Nerve Anatomical Connections and Their Clinical Value: A Narrative Review
Source: Diagnostics (Basel). 2026 Jun 15;16(12):1855. doi: 10.3390/diagnostics16121855 (PMC13298086; doi:10.3390/diagnostics16121855)
Supplement: Supplementary file 1 [file diagnostics-16-01855-s001.zip › diagnostics-4296345-supplementary.pdf]

Table S1. Comprehensive Anatomical Map of Peripheral Trigeminal–Facial Nerve Communications. Table compiled from Cobo et al. [1, 3], Diamond et al. [2], Hwang et al. [8, 9], Yang et al. [13], Tansatit et al. [10], Iwanaga et al. [26], Iwai et al. [18], Mohanty et al. [14], Kwak et al. [7], Namking et al. [6], Shimada et al. [11], and Haroun [4]. Abbreviations: ATN = auriculotemporal nerve; BB = buccal branch (CN VII); BN = buccal / buccinator nerve (V3); CATN = communicating auriculotemporal nerve; CBr = cervical branch (CN VII); CT = chorda tympani; FT = facial trunk; GAN = great auricular nerve; ION = infraorbital nerve (V2); ITF = infratemporal fossa; ITN = infratrochlear nerve (V1); LN = LN (V3); LLSAN = levator labii superioris alaeque nasi; LODiv. = latero-orbital (zygomatic) division [13]; MMBr = marginal mandibular branch (CN VII); MN = mental nerve (V3); MODiv. = medio-orbital (nasion) division [13]; OGng = otic ganglion; SCDiv. = superciliary (supraorbital) division [13]; SMGl = submandibular gland; SLGl = sublingual gland; SON = supraorbital nerve (V1); STN = supratrochlear nerve (V1); TB = temporal branch (CN VII); TCN = transverse cervical nerve; TFD = temporofacial division (CN VII); TH = tyrosine hydroxylase; VAChT = vesicular acetylcholine transporter; ZB = zygomatic branch (CN VII); ZFN = zygomaticofacial nerve (V2); ZTN = zygomaticotemporal nerve (V2).

Panel A. Trunk-Level and Intraparotid Communications

Trigeminal / Cervical Branch	CN VII Target	Prevalence (Cadaveric)	Prevalence (Sihler)	No. of Communicating Branches	Connective-Tissue Architecture	Topographic Depth / Location	Key References
ATN (V3)	FT / TFD	93.3%	100%	2–4 per specimen; 3-branch pattern commonest (46.7%); single branch never observed	Not histologically characterised	Deep within parotid substance; CATN arises near root of auriculotemporal n.	[6, 7, 18]
GAN	FT	100%	100%	Not specified	Not histologically characterised	Within parotid gland; extracranial cervical plexus contribution	[1, 25]

Panel B. Upper Face (Forehead / Periorbital Region)

Trigeminal Branch (Division)	CN VII Target	Prevalence (Cadaveric)	Prevalence (Sihler)	Topographic Zone [13]	Connective-Tissue Architecture	Target Muscles	Key References
SON horiz.br. (V1)	TB	44%	85.7% (12/14)	SCDiv.	Common epineurium, separate perineuria	Frontalis, procerus, depressor supercilii, corrugator supercilii	[13, 23]

STN (V1)	TB	—	57.1% (8/14)	Superciliary div.	Not characterised	Corrugator supercilii, orbicularis oculi	[13]
STN (V1)	BB (long asc.)	—	50% (7/14)	MODiv.	Not characterised	Orbicularis oculi, corrugator supercilii (at origin)	[13]
ITN (V1)	BB (long asc.)	—	85.7% (12/14)	MODiv.	Not characterised	Orbicularis oculi, procerus	[13]
ZTN (V2)	TB	Variable	Overlap only; no definite fibre exchange	LODiv.	Not characterised; may carry parasympathetic fibres to lacrimal gland	Frontalis, orbicularis oculi (lateral)	[11, 27]
ATN (V3)	TB	Present	—	Preauric./temp.	Not characterised	Frontalis, orbicularis oculi	[1]

Panel C. Midface (Zygomatic / Buccal / Infraorbital Region)

**Trigeminal Branch (Division)**	**CN VII Target**	**Prevalence (Cadaveric)**	**Prevalence (Sihler)**	**Communication Pattern**	**Connective-Tissue Architecture**	**Target Muscles**	**Key References**
ZFN (V2)	ZB	—	41.7–85.7%	Single connection	Not characterised	Orbicularis oculi (lateral), zygomaticus major/minor, LLSAN	[11, 13]
ZFN (V2)	BB	—	41.7%	Single connection	Not characterised	Zygomaticus major, levator anguli oris	[13]

ION (V2)	BB	Up to 96%	100% (Yang); 50% (Iwai, strict)	Triple (25%), double (62.5%), single (10%); most common: lower buccal trunk ↔ lateral labial br. of ION (70%)	**Common epineurium AND common perineurium** — most intimate plexus in face	Zygomaticus major/minor, levator labii superioris, buccinator, orbicularis oris	[8, 10, 13, 18]
ION (V2)	ZB	—	28.6% (4/14)	Tiny twigs only	Not characterised	Orbicularis oculi	[13]
BN (V3)	BB	~100%	100% (Iwai)	Thick anastomotic branch at deep fascia of buccinator or within buccinator/orbicularis oris	VAChT gradient confirms gradual sensory→motor fibre transition [14]	Buccinator, orbicularis oris, risorius	[11, 14, 18]
BN (V3)	ZB	Present	—	—	Not characterised	Orbicularis oculi, zygomaticus major/minor	[2]
STN (V1)	ZB	—	50%	—	Not characterised	Orbicularis oculi	[13]

Panel D. Lower Face (Mandibular / Mental Region)

Trigeminal Branch (Division)	CN VII Target	Prevalence (Cadaveric)	Prevalence (Sihler)	Morphometric Details	Connective-Tissue Architecture	Target Muscles	Key References
MN (V3)	MMBr	100% (23/23)	50% (Iwai, strict)	Mean 8.26 ± 2.49 connection sites per specimen within 6 × 12 mm rectangle; two types: "superior" (to lower lip, 70%)	Common epineurium, **separate perineuria** [24, 26]	Orbicularis oris, depressor anguli oris, depressor labii inferioris, mentalis	[18, 24, 26]

				and "anterior" (to chin, 100%); mean diameter 0.4 mm, 1–8 branches (mean 2.6) per side			
MN (V3)	BB	Present	—	—	Not characterised	Orbicularis oris, depressor labii inferioris	[11]
BN (V3)	MMBr	Present	—	—	Not characterised	Orbicularis oris, mentalis	[1]

Panel E. Cervical Region

Trigeminal / Cervical Branch	CN VII Target	Prevalence	Target Muscles	Key References
TCN	MMBr	~24.7%	Platysma, depressor anguli oris	[35]
CBr ↔ MMBr (intrinsic CN VII)	—	~24.7%	Platysma	[1]

Panel F. Deep (Intracranial / Infratemporal) Communications

CN VII Branch	CN V Target	Prevalence	Fibre Types Conveyed	Functional Significance	Key References

CT	LN (V3)	100% (constant; embryologically at 18 mm stage)	Special sensory (taste, anterior 2/3 tongue) + preganglionic parasympathetic (SMGl & SLGl)	LN injury disrupts both V3 somatosensory and CN VII taste/secretomotor fibres simultaneously	[2, 37, 41]
CT	OGng (via ITF)	Constant	Parasympathetic fibres to parotid gland (via CN IX pathway)	Creates CN VII–CN IX functional link; implicated in Frey syndrome pathophysiology	[44]

Panel G. Summary of Prevalence by Trigeminal Division and Method

Trigeminal Division	Cadaveric Dissection ([9] pooled)	Sihler Staining [13]	Sihler Staining — Strict Criteria [18]
**V1 (Ophthalmic)**	33.8% ± 19.5%	85.7%	0% (crossing only, no fibre exchange)
**V2 (Maxillary)**	95.0% ± 8.0%	100%	50% (via infraorbital n.)
**V3 (Mandibular)**	76.7% ± 38.5%	100%	100% (auriculote

mporal + buccal n.); 50% (mental n.)

Panel H. Connective-Tissue Architecture by Facial Region

Facial Region	CN V Branch	CN VII Branch	Epineurium	Perineurium	Degree of Fibre Intermingling	Reference
Forehead	SON (V1)	TB	Common (fused)	**Separate**	Lower — fibres travel together but maintain fascicular identity	[23]
Midface	ION (V2)	BB	Common (fused)	**Common (fused)**	**Highest** — most intimate sensorimotor plexus	[8]
Chin	MN (V3)	MMBr	Common (fused)	**Separate**	Lower — pattern mirrors forehead	[24]
Buccinator zone	BN (V3)	BB	Common (fused)	VAChT gradient confirms fibre exchange	High — gradual motor↔sensory transition confirmed immunohistochemically	[14]
